# Community engagement in pastoralist areas: Lessons from the public dialogue process for a new refugee settlement in Turkana, Kenya

**DOI:** 10.1186/s13570-021-00192-7

**Published:** 2021-11-24

**Authors:** Cory Rodgers

**Affiliations:** Refugee Studies Centre, Oxford Department of International Development, University of Oxford, Oxford, UK

**Keywords:** Pastoralism, Kenya, Land, Elites, Public participation, Community dialogue

## Abstract

Large-scale development interventions have long failed to accommodate the needs and preferences of pastoralists or the systems of resource governance and land tenure upon which they rely. However, advocates of rights-based approaches to development emphasise the importance of community participation in planning and agenda-setting, and in Kenya, public participation is a formal constitutional requirement for government decision-making processes. In 2015, the United Nations High Commissioner for Refugees concluded negotiations with local stakeholders about the use of 15 km^2^ of communal rangelands to build a new refugee settlement in Turkana County, Kenya. Negotiations entailed a community dialogue process involving local people living in the vicinity of the proposed settlement. This paper retrospectively examines the inclusivity of the dialogue process, with particular attention to the involvement of pastoralists and the representation of their interests. Interviews and focus groups conducted with a range of key informants and community stakeholders highlighted two key problems. First, negotiations relied upon a simplistic approach to communal land tenure that overlooked the complexity of overlapping and often contested access rights. Second, there was an over-reliance on urban professionals and politicians as intermediaries between rural communities and development actors. Even where elite intermediaries act in good faith, they may introduce an ‘oppidan bias’ into development policies, thereby marginalising the viewpoints of non-urban, non-sedentary demographics, such as pastoralists. I conclude with recommendations for the UNHCR to develop a more explicit strategy for direct engagement with host community stakeholders in Turkana and with increased attention to the interests of livestock producers and the nuances of pastoralist land use.

## Introduction

Pastoralists sustain their herds by exploiting spatially and temporally variable forage, and they require physical mobility and communal rights to access territory and water sources (African Union 2010). But prevailing models of development often contribute to territorial enclosure, resource privatisation and more permanent forms of settlement (Galvin 2009; Tilahun et al. 2016). This is especially acute in the case of large-scale projects involving high levels of investment and expansive land acquisitions, often for the purposes of commercial agriculture, industrial resource extraction, infrastructural construction and urban expansion (Lind et al. 2020). Moreover, international law has often failed to provide adequate protection for pastoralists’ land rights, in part because legal conventions are poorly equipped to deal with complex land tenure systems that involve communal access and seasonality of use (Gilbert 2012).

When development projects are undertaken in areas inhabited by pastoralists, community engagement is a crucial—albeit complicated—process (Herrera et al. 2014: 4). Where pastoralists are the target ‘beneficiaries’ of development projects, community engagement can serve programme objectives by bringing local knowledge to bear on project design (Flintan and Cullis 2010, Mangesho et al. 2017), as well as to build trust and improve uptake of services amongst targeted communities (Okello et al. 2014; Caudell et al. 2019; Griffith et al. 2020). But in the context of large-scale development projects, pastoralists instead find themselves in the less enviable role of an ‘affected community’, where land dispossession, environmental degradation and various other harms are the collateral damage of development (Behnke and Kerven 2013; Gabbert 2021). Thus, community engagement is both a means to the end of making development programmes more attentive to local knowledge *and* an end in itself, i.e. ensuring pastoralist involvement in democratic processes (Morton 2010: 8). Participation is also necessary to support the adaptive governance of customary institutions themselves (Folke et al. 2005: 449). It is through sustained public discourse that ‘local knowledge’ is regenerated in response to changing circumstances (Spencer 2004: 211–212). When customary institutions are plugged into multi-level systems involving formal governmental agencies and NGOs, they gain access to new forms of knowledge that can contribute to their adaptive capacity (Robinson and Berke 2011: 1193). While dismissive or antagonistic state activity may weaken customary institutions (Schmidt and Pearson 2016: 29), cooperative arrangements with state actors can ensure that customary institutions remain active and relevant.

The necessity of community engagement is grounded in the ‘duty to consult’ (Ayele 2015: 280; Gebeye 2016: 5) and the need for ‘Free, Prior, Informed Consent’ (LaTosky 2021), as well as principles such as self-determination that are at the core of the rights-based approach to development (Gilbert 2012; Bassi 2017). Without mechanisms to negotiate the terms of development, pastoralist communities are rendered voiceless and without means to ensure that their interests are represented. Nonetheless, development is often undertaken without the involvement of pastoralists or recognition of their knowledge and expertise (Abbink et al. 2014). In some contexts, pastoralism has been seen as a barrier to progress, and its eradiation has been an explicit aim of policy (Gebeye 2016). Even where this is not the case, pastoralist peoples have often been politically and economically marginalised in state policy (Pavanello 2009: 8), which makes participatory processes especially difficult (Herrera et al. 2014: 4). Moreover, identifying the appropriate pastoralist ‘community’ is complicated by the co-existence of multiple groups with overlapping governance structures and contested claims to land and resources (Cormack and Kurewa 2018).

This article provides an in-depth case study of the community engagement conducted by the United Nations High Commissioner for Refugees (UNHCR)—otherwise known as the UN Refugee Agency—in order to establish a new refugee settlement in north-western Turkana County, Kenya. While refugee operations are generally seen as a humanitarian rather than a development issue, the establishment of the Kalobeyei Settlement evades this dichotomy. The design of the Kalobeyei Settlement was based on the idea of a humanitarian-development ‘nexus’, wherein funding for refugee protection is leveraged to promote regional development goals such as economic growth and provision of social services and infrastructure (UNHCR 2018). The project has also required some of the potential harms that are typical of large-scale development interventions; in particular, the construction of the Kalobeyei Settlement required a land acquisition of about 15 km^2^ from the local population, many of whom rely heavily on mobile livestock-keeping for subsistence.

As explained on the ‘Kalobeyei Settlement’ page of the UNHCR website, ‘during the World Refugee Day commemoration held in Kakuma on 20 June 2015, the land was officially handed over by the County Government *and people* of Turkana’ (UNHCR 2020a, emphasis mine). By reviewing the community engagement processes that preceded this event, I critically examine the claim that ‘the people’ handed over their land in Kalobeyei. Not all community engagement processes ensure that communities are able to participate efficaciously; some approaches serve as a means to co-opt community voices into pre-determined agendas (Kothari 2001; LaTosky 2021). As Herrera et al. write in regard to rangeland governance, ‘participation in decision-making cannot be developed without properly addressing questions like capacity building, equity, voice, empowerment, gender or transparency’ (Herrera et al. 2014: 4).

In Kenya, ‘public participation’ in government decision-making is a formal constitutional requirement. Article 61 gives the public a say in decisions about land, and Article 69 requires the state to ‘encourage public participation in the management, protection and conservation of the environment’ (Government of Kenya 2010). However, public participation processes in Kenya have often been undertaken as ‘rubber stamp’ activities, with poor communication of information to the public, limited attendance and political rather than independent oversight (Nyaranga 2019).

Where formal channels for public participation and community engagement are inadequate or where institutional trust is insufficient, communities make their demands known in other ways. Refugee operations in Turkana provide a case in point. The UNHCR has made efforts in recent years to become less refugee-centric and to attend to the well-being of host communities affected by the arrival of forced migrants (Rodgers 2020c). In part, this is an expression of solidarity with communities that face the burden of hosting large refugee populations. But it is also a defensive response to a threatening political reality. UNHCR’s ability to protect refugees in Turkana has at times been compromised by violence from disgruntled locals (Crisp 2000), who demand compensation for the burdens that they face as a host community (Aukot 2003). In regard to the political agency of the local population in Kakuma, one UNCHR evaluation remarked that the UNHCR ‘is aware of their plight and, in a worst-case scenario, vulnerable to their threats’ (Jamal 2000: 29). Host community engagement is therefore crucial to UNHCR’s ability to provide protection to refugees under its mandate.

Elsewhere in Kenya and eastern Africa more generally, large-scale projects in the drylands are simultaneously welcomed as an investment to address historical marginalisation and protested as a threat to the livelihoods, land rights and cultural heritage of pastoralists (Cormack 2016; Enns 2019). Negotiations between external organisations and local communities are complicated by political contestations *within* communities, as elites mobilise different groups to stake contested claims for compensation and benefits (Lind et al. 2020). Particularly useful lessons can be drawn from the extractive activities that have taken place in southern Turkana. When the Anglo-Irish firm Tullow Oil discovered oil in 2012, the national government’s optimism was countered by stark warnings of a ‘resource curse’, in which struggles to profit from the oil would incite violent conflict between disenfranchised locals and external governmental and corporate stakeholders (Johannes et al. 2015). While overt violence has thus far been limited, local people have forcefully demanded jobs and benefits through protests, road blocks and occupations of company sites (Schilling et al. 2015). In light of public participation requirements and in pursuit of local legitimacy, Tullow Oil has put in place extensive community engagement mechanisms, including hiring of community liaison officers, large town halls meetings with local residents and consultations with specific demographics such as elders and women’s groups (Okenwa 2020).

Aside from direct community engagement, Tullow Oil has also relied on mediation by the Turkana County Government (TCG). The discovery of oil in 2012 roughly coincided with the process of devolution, which was one of the most significant outcomes of the promulgation of a new national constitution in 2010. Devolution entailed a major reorganisation of state structures that passed an array of powers from the central government to 47 newly created county governments, which was executed in 2013. One of the intentions of this reform was to shift decision-making to a more locally accountable layer of government (Kanyinga 2016).

However, devolution has also had the unintended effect of exacerbating tensions at the sub-national level (Lind 2018). While Tullow reports spending over 2.5 million USD on local corporate social responsibility, the advocacy organisation Sustainable Approaches for Community Empowerment (SAPCONE) alleges that the company’s reliance on local leaders with political ambitions has led to misuse of funds intended for community projects (Waruru 2019). During negotiations for community benefits from oil extraction, county-level politicians in Turkana have been accused of dominating discussions and preventing effective engagement with the broader community, district advisory committees have allegedly been affected by nepotism and corruption, and community liaison officers are often seen as loyal to their employer (the Tullow Oil company) rather than the communities with whom they engage (Mkutu et al. 2019; Okenwa 2020). As indicated in Tullow’s stakeholder engagement plan, government intermediaries cannot completely replace a more inclusive dialogue process:

While it is clear that County Government and National Government officials are key representatives of pastoralists, there are other traditional structures that exist and need to receive an opportunity to receive information and give feedback. Experience has clearly shown that while County and National officials have direct lines of contact with traditional leaders, some traditional leaders may have felt excluded. (Golder Associates Ltd. 2020: 12–13)

Moreover, Turkana residents living in the vicinity of the extraction sites have foregone government mediation to engage the company directly, attempting to ‘stretch the mandate’ for benefit-sharing (Okenwa 2020: 62). Long accustomed to marginalisation in national politics, they have exercised a form of ‘crude citizenship’ that puts the company in the conventional role of a state (Enns and Bersaglio 2015).

This article offers a similarly critical account of the community engagement process in UNHCR’s refugee operations in north-western Turkana. This study is not a legal analysis, and it does not attempt to determine whether or not the minimum standards for public participation were met, as I do not possess the required legal expertise. Rather, I draw on focus group discussions and individual interviews with people involved in or affected by the construction of the Kalobeyei Settlement to provide an anthropological analysis of the perceived inclusivity and legitimacy of the community engagement process that preceded the handover of the land. Unlike Tullow Oil, the UNHCR has not published an explicit strategy for host community engagement. While UNHCR has collaborated with the TCG to develop an extensive Kalobeyei Socio-Economic Development Plan (KISEDP), this plan does not detail structures and mechanisms for facilitating public participation and dialogue. In practice, the UNHCR’s community engagement relies heavily on mediation by national and local government authorities. This case study suggests that this approach is resulting in an ‘oppidan bias’ in policies and programmes and leaving many people feeling disillusioned or left out, especially those who rely primarily on pastoralist production.

## Study area

### Geographical context

Turkana, the largest of Kenya’s 47 counties, is located along the country's north-western borders with Uganda, South Sudan and Ethiopia. It is bordered on the east by Lake Turkana, and much of the county is located within an arid lake basin, with cooler escarpments rising to the south, west and northwest. Most of the population identify as *Ng’iturkana* (Turkana people) and speak a language similar to that of their Karamojong, Dodos, and Jie neighbours in Uganda, as well as Toposa in South Sudan and Nyang’atom in Ethiopia. The Turkana basin is largely constituted by arid plains that receive  long rains between March and April and lighter rains starting in September or October. Primary food production relies heavily on pastoralism, with herds composed of cattle, camels, goats, sheep and donkeys. While census data does not identify ‘pastoralists’ as a category, a review of three alternative datasets available suggests that about 50% of the population in Turkana depend on livestock for their livelihood (Krätli and Swift 2014). Rural herders supplement their livelihoods with seasonal sorghum cultivation, hunting and forest resources. Development projects have promoted irrigated agriculture near some rivers and fishing along the lake, while the growing towns and settlements support a modest labour market.

**Fig. 1:**
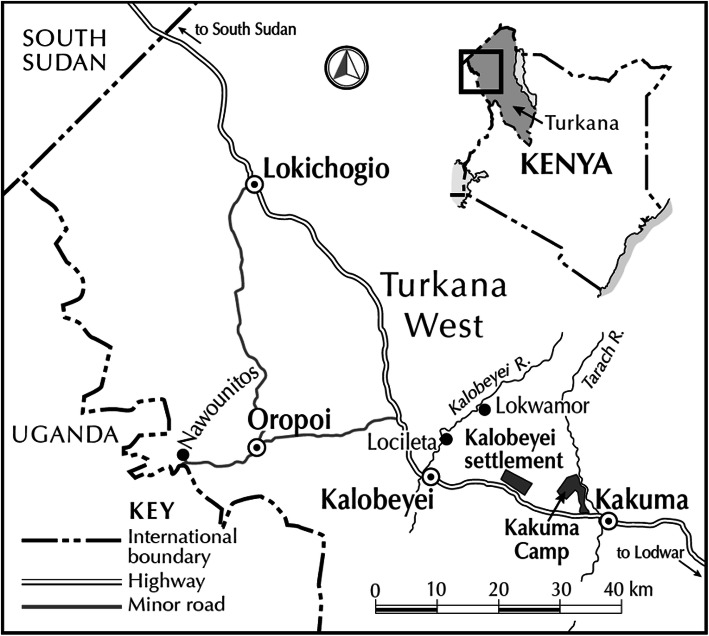
Map of relevant sites in Turkana West sub-County. Coordinates of major centres and refugee settlements are publicly available, while coordinates of smaller settlements and rural locations were recorded during fieldwork using phone-based GPS. (Produced by John Hall, 2020, at the author’s request)

From the county’s centrally located capital of Lodwar, a highway stretches for 250 km northward to the border with South Sudan. Kakuma town is at the halfway point of this journey, where the Tarac River crosses the highway (see map in Fig. 1). The town occupies most of the eastern side of the river, and the Kakuma refugee camp stretches out along its western side. About 17 km further along the highway is the access road to the more recently constructed Kalobeyei Settlement. The settlement shares its name with Kalobeyei Town, located another 15 km up the highway. Under the county government system formed after devolution, both Kakuma and Kalobeyei are recognised as distinct wards, each with its own representative in the County Assembly. Parallel to this, the national government continues to recognise elements of the older provincial system, which were preserved in the 2013 National Government Coordination Act and which designate Kakuma and Kalobeyei as *locations* with their own chiefs appointed by the Office of the President.

### Historical context

Turkana has long existed at the margins of national development in Kenya. In the early twentieth century, the British colonial government underook a series of punitive raids against Turkana militias and their Abyssinian supporters. Afterward, they treated Turkana as a ‘closed’ district with limited investments in administrative or development activities (McCabe 1994). Following national independence in 1963, the Government of Kenya made modest investments in roads, irrigation, fishing and urban infrastructure (Hogg 1987). However, Turkana received disproportionately low levels of investment in the national development agenda (Oxfam 2006), which contributed to a growing sense of marginalisation amongst Turkana people (Aukot 2003).

In 1992, a camp was established in Kakuma after tens of thousands of refugees from southern Sudan—who had been gathered in the border town of Lokicogio to the north—were relocated by the UNHCR. Their arrival was precipitated by a regime change in Ethiopia; southern Sudanese rebels had been operating out of Ethiopia’s Gambella Region under the socialist DERG government, but when the Ethiopian People’s Revolutionary Democratic Front—which was friendly with the Sudanese government—came to power, the rebel militias were ejected along with approximately 150,000 Sudanese refugees. They returned to south-eastern Sudan but were soon routed by the Sudanese military, after which they turned toward Kenya (Ohta 2005).

Prior to the establishment of the Kakuma camp, Kakuma town was a small settlement of between 2000 and 8000 people, with a larger semi-nomadic population living in the surrounding plains. For many pastoralists, emerging urban areas like Kakuma were seen as spaces of destitution, rather than ‘progress’ (Broch-Due and Sanders 1999). But the influx of refugee aid created many economic opportunities in Kakuma, fostering domestic immigration from elsewhere in Turkana as well as other parts of Kenya. It has also been a cause of tension and occasional violence. Accustomed to a nearly homogeneous ethnoscape—with the exception of a few European missionaries and Kenyan traders—Turkana people suddenly found themselves living amongst a growing and increasingly diverse population (Ohta 2005). Some Turkana elders attributed an increase in armed robberies to the presence of refugees and the influx of firearms that they brought with them (Aukot 2003). Moreover, when Sudanese refugees arrived with their own livestock, the animals were often subject to confiscation by Turkana locals, occasionally leading to clashes (Crisp 2000).

Land has been an especially prominent source of tension. When the Kakuma camp was first constructed, land was allocated to the UNHCR by the central government, in a process that largely excluded local residents. At the time, most of the land in Turkana was ‘Trust land’ under the management of local county councils, who had little power to oppose the actions of the central government (Future Agricultures 2014). The land on which Kakuma was built was formerly used for wet season grazing, and the surrounding areas were rapidly depleted of vegetation as the growing camp population procured firewood for their cooking fires. A socio-economic assessment by the World Bank shows that while the refugee presence has boosted some economic activities, the impact on livestock production has been negative (Sanghi et al. 2016: 53). Moreover, the UNHCR compound in Kakuma, a walled-off complex containing offices and residences for humanitarian staff and official visitors to the camp, stands on fertile ground where some local residents used to plant seasonal sorghum gardens (*ng'amanat*). Many local Turkana still refer to the camp as ‘our soil’ (*ng’alup nakosi*), and refugees refer to the host population—somewhat flippantly—as ‘our landlords’.

Prior to the land acquisition process described below, the Kakuma camp had already been expanded on several occasions to accommodate a growing refugee population. However, the promulgation of a new constitution in 2010 created a new regulatory regime for land. All former Trust lands were to be converted to ‘Community land’ registered to specific communities. Until such registration could take place—which required a Community Land Bill that was not passed until 2016—the ex-Trust lands would be managed by the newly formed County Governments, which were expected to be more attentive than central government to the needs and interests of local constituents (Boone et al. 2016). This new arrangement set the stage for the community dialogues discussed here.

## Methods

My understanding of the socio-economic context for this study draws on a cumulative two-plus years of ethnographic fieldwork in Turkana County, where I began my doctoral research in January 2015. For this study, I draw particularly on 1 month of fieldwork in July 2016, 2 months in August and September 2017, 1 month in December 2018 and 2 weeks in December 2019. During these periods, I conducted 8 focus group discussions (FGDs) targetting communities at varying proximity to the refugee settlements and covering different socio-economic profiles (see Table 1). Recruitment was conducted immediately prior to discussions; the aims of the study were explained, willing participants gathered in a designated spot and consent was requested verbally. If attendees agreed, the conversation was recorded for later transcription.

**Table 1:** Focus group discussions

	Location	Timing	Attendee profiles
1	Natukobenyo	July 2016	Approximately 10 rural residents, both women and men, mostly working in construction, petty trade and food service in the settlement; some tend small herds of goats
2	Naabek	July 2016	8 peri-urban village residents, 4 women and 4 men, living adjacent to the Kakuma camp, employed as domestic workers or porters by refugee businesses/households
3	Natukobenyo	August 2017	Approximately 15 rural residents providing labour or selling goods to refugees in the settlement; some tend small herds of goats
4	Nalemsekon	August 2017	Large gathering of 30 to 40 peri-urban village residents, both women and men, living adjacent to the Kakuma camp, many employed by refugee businesses/households
5	Kalobeyei	August 2017	Approximately 10 urban residents in Kalobeyei Town; mostly young men
6	Lokwamor	August 2017	Approximately 15 women and men from households tending herds of goats north of Kalobeyei Town
7	Oropoi	September 2017	Six town residents, 4 women and 2 men, living in the Oropoi settlement 40 km west of Kalobeyei Town
8	Nawounitos	September 2017	8 pastoralists, all men, tending cattle far to the west of Kalobeyei

Discussions focused on the impact of refugee operations on the Turkana ‘host community’, relations between locals and refugees, and expectations about benefits associated with the new settlement. FGD findings were cross-checked during key informant interviews with local government officials, high-level employees of humanitarian organisations and residents of Kakuma and Kalobeyei, some of which were conducted remotely by phone during the Covid-19 pandemic (see Table 2). FGDs and interviews were carried out by the author in English, Swahili and Turkana according to the linguistic preferences and abilities of the group. A research assistant who spoke Turkana as his first language was on hand to assist with difficult translations or points of misunderstanding.

**Table 2:** Individual interviews

1	Kalobeyei interviews	December 2018	11 women and 1 man living on the periphery of Kalobeyei Settlement, most involved in petty trade and goat husbandry
2	Kakuma interviews	December 2018	10 women and 1 man living near the Kakuma camp, most involved in petty trade and domestic work
3	Agency interviews	2016–2019	Various humanitarian workers employed by the UNHCR, World Food Programme (WFP), Lutheran World Federation (LWF), UN-Habitat and Norwegian Refugee Council (NRC)
4	Phone interviews	October 2020	2 former members of the Community Development and Dialogue Committee (see below), both men from Kalobeyei. One served as the chairman of the committee, and the other was the liaison officer for the UNHCR

Rather than coding all transcripts together at the end of the study, data collection and analysis were combined in an iterative process, with each discussion structured according to findings from previous discussions. This allowed for confirmation of the sequence of historical events, elicitation of differences in perspective based on livelihood and location, and in-depth probing of contested topics.

## Results

### Handover of land in Natukobenyo

**Fig. 2:**
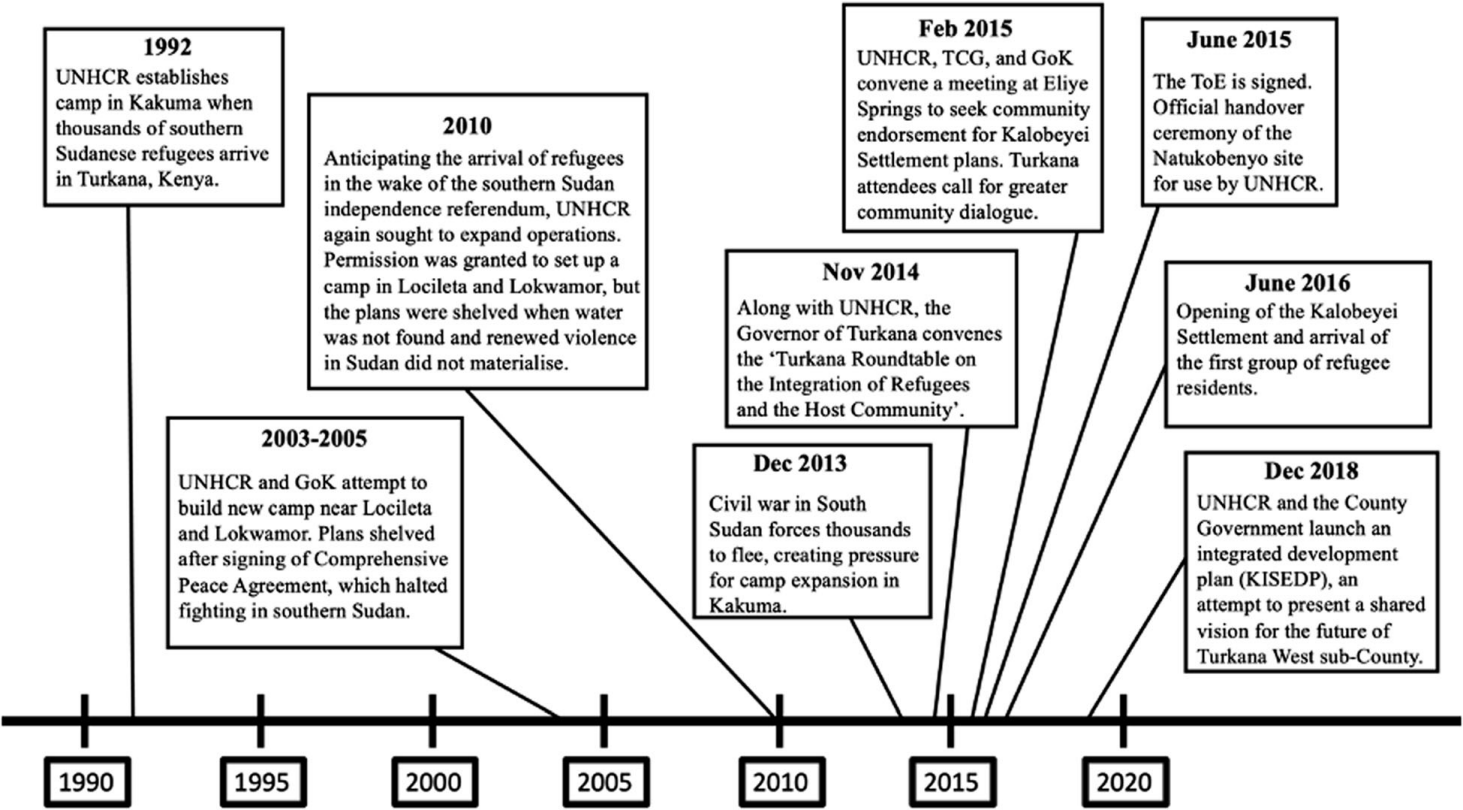
Timeline of key events in the establishment of the Kalobeyei Settlement. This timeline was assembled based on input from both FGDs and interviews as well as a desk review of relevant reports, especially the Terms of Engagement (discussed below) and the Kalobeyei Settlement Advisory Development Plan produced by UN-Habitat (2018). There is some disagreement with the Terms of Engagement document, which is dated to the opening (February 2015) of a dialogue that lasted multiple months, rather than the date when the agreement was signed

As shown in Fig. 2, UNHCR has on several occasions attempted to access additional land for expanding refugee operations, first in 2003 and again in 2010. In both cases, negotiations focused on an area in the vicinity of Lokwamor and Locileta, located along the Kalobeyei River and about 20 km west of Kakuma. This site was suitable for a camp due to the presumed availability of groundwater from the river. However, Locileta and Lokwamor were also important wet season grazing areas for pastoralists, who have historically returned to the area with their cattle and goats after enduring the dry season in the more dangerous borderlands further west. Reflecting on these events, the Chief of Kalobeyei Town recalled that the government expressed an intention to negotiate, but wasted no time surveying the site and starting construction.

At one point – and it was unclear whether this followed the 2003-05 or 2010 attempt to gain access to the Locileta and Lokwamor sites – men gathered in Lokwamor to perform an *agata*, a collective chanting ritual associated more with rural traditional life than urban modernity. The *agata* is performed by male elders on various occasions, including weddings, initiation rites for youth, and at gatherings convened by an *emuron* (a recognised seer or diviner) to deal with community-wide problems such as drought or insecurity. The intention of the chanting is to impose a collective will upon the land, to bring order and coolness to tense situations and to chase out undesirable spirits, human or otherwise. Since that time, various failures and accidents have been attributed to the *agata* ceremony.

The old men did some curses on that land. Then, when people from UNHCR went to demarcate that land and drill water, something mysterious happened to them. Their truck sank into the soil… They failed to get water in Lokwamor because the old men of the area had cursed the land with those rituals. The engineers confirmed that there is water there, yet all the boreholes were dry! (Urban Resident, Kalobeyei Town, September 2016)

The *agata* was allegedly accompanied by acts of sabotage, including the removal of survey beacons and refilling of freshly dug latrine pits. In hindsight, some locals have seen these protests as successful, because no camp was ever actually established in Lokwamor and Locileta. Of course, there were other factors at play, as events in Sudan reduced the apparent need for camp expansion. In 2005, the signing of the Comprehensive Peace Agreement allowed many refugees to return to southern Sudan, significantly reducing the camp population. And again in 2011, the completion of the South Sudanese independence referendum seemed to avert another wave of displacement, thereby diminishing the pressure for camp expansion.

As of November 2013, over 127,000 refugees—most from Somalia and South Sudan—lived in the camp. That December, the start of the civil war in South Sudan forced many people to seek asylum in Uganda and Kenya. The influx of new arrivals quickly exceeded the capacity of the Kakuma camp, even as new shelters were constructed in an expanded section named ‘Kakuma 4’. In order to decongest Kakuma, the UNHCR reached out to the government to find additional territory.

However, as described above, the terms for acquiring access to land had changed with the passing of the new constitution in 2010. Rather than simply designating new territory for the settlement, a process of community engagement was convened by the National and County governments, as well as UNHCR. This process relied heavily on local ‘elites’, a term that refers to urban, educated individuals who draw influence from their connections to government and NGOs. After several failed attempts to secure access to land in Lokicoggio and Nakoyo, and recognising the persistent community opposition in Lokwamor and Locileta, some of the local elites from Kalobeyei directed the government to Natukobenyo, a site located about midway between Kakuma Town and the Kalobeyei River (see Fig. 1 map).

In February 2015, the national and county governments selected a group of elites from Kalobeyei and invited them for a meeting at Eliye Springs, a resort on the western shore of Lake Turkana. However, news of the meeting was disseminated throughout their networks, and the turnout was larger than expected. One of the uninvited attendees commented:

They [government and UNHCR] said they were coming to negotiate. In a real sense, they were coming to get an endorsement for something they had already decided at the leadership level. But when we attended and spoke up as professionals, we altered the process. (Kalobeyei Resident, Phone Interview, October 2020)

Attendees called for a more comprehensive process of community outreach. A group of professionals from Kalobeyei formed a 7-member Community Dialogue & Development Committee (CDDC), with the objective of speaking to people throughout Turkana West sub-County about the plans for the Natukobenyo site. The UNHCR appointed one member of the committee as a Community Liaison Officer, whose job was to communicate CDDC findings to the UNHCR and its partners. During the months that followed, the CDDC reached out to people across Turkana West. Discussions focused on the environmental and social impact of encampment, as well as host community complaints about access to water, health services, education and livelihoods. The CDDC used their research to inform negotiations with the UNHCR and the TCG, and an agreement was finally reached in June 2015. A document titled ‘Terms of Engagement for a 2^nd^ Refugee Camp in Kalobeyei’ (ToE) was signed by representatives of the County and National governments, as well as UNHCR, and the Natukobenyo site was officially handed over to the UNHCR on World Refugee Day in June 2015. Relocation of refugees to the new settlement began in May 2016 and continued until June 2017, when the settlement population reached maximum capacity at nearly 40,000 people.

### Barriers to local legitimacy

The ToE is the product of a dedicated effort to engage the local community in dialogue about the UNHCR’s refugee operations. In it, the CDDC put forward a long list of demands that the UNHCR agreed to incorporate into their plans for the Kalobeyei Settlement, including provision of employment to local people, the award of contracts and tenders to local businesses, and support for education and health services. A number of points focused specifically on support for pastoralism:

- Provision of water for livestock, using dams, rock catchments, water pans, etc.
- Support for livestock production, health and husbandry
- Destocking and restocking programmes at appropriate seasons
- Protection of indigenous knowledge, bio-diversity and other resources
- Preservation and protection of dignity of cultural practices and traditions

However, interviews and focus groups conducted between 2016 and 2018 yielded many complaints that some locals had been excluded from the process and that their needs and expectations were not being met in practice. As described elsewhere (Rodgers 2020a), those engaged in pastoralism were especially disappointed.

It is important to qualify this claim with three caveats. First, the Kalobeyei ‘project’ is still only in early stages, and it is too early to make claims about overall success or failure. Second, not everyone is dissatisfied. Many entrepreneurs have built shops in the settlement. A handful of Turkana people living in the direct vicinity of the Settlement are enjoying improved access to health services, close access to well-stocked shops, and a market of refugee customers to whom they could sell firewood or locally produced charcoal. Some even received free stone houses constructed under a UNHCR cash-for-shelter programme. And finally, implementation has faced some unexpected complications. The original plan for an integrated settlement aimed at a ‘hybrid community’, in which refugees and Turkana ‘hosts’ would live together and share integrated services—schools, clinics, water utilities, etc. However, few Turkana people were willing to move into the settlement, for fear of leaving their existing neighbourhood networks and living in a place where they felt like a minority. According to teachers at the Morning Star Primary School in the settlement, by 2019 there were over 3000 refugee students but only about 10 pupils from the host community. One response was that the UNHCR widened the scope of its partnership with the TCG. Rather than restricting the project to the 15 km^2^ of the settlement, the partnership evolved into the Kalobeyei Integrated Social and Economic Development Plan (KISEDP). This more ambitious agenda encompassed all 1500 km^2^ of Turkana West sub-County, but because it no longer entailed co-residence of refugee and host communities in the settlement, integrated service delivery was complicated.

When reviewing the preliminary findings of my research with friends in Turkana, some expressed scepticism about the complaints that I was hearing. A common remark—including amongst people who were themselves Turkana—was that rural leaders have a cultural predilection during public meetings to negotiate for more, regardless of what has already been received. Others suggested that the oil negotiations in the south had set a precedent that politicised the dialogue process in Kalobeyei, creating unrealistic expectations. 

Nonetheless, interviews and FGDs conducted for this study encouraged discussants to elaborate upon their complaints. The sections ahead consider some of the specific reasons that certain individuals or groups were left to feel disenfranchised by the structure of the community engagement process. They reveal that despite efforts at inclusivity by the UNHCR and its partners, the community dialogue process was compromised by poor understanding of pastoral land use and heavy reliance on urban mediators.

#### Contested claims to communal land

As described above, the CDDC prioritised geographical coverage in its approach to community dialogue. The membership of the CDDC was comprised of two to three members from each ward or location in Turkana West sub-County. Individuals would speak with people in their respective locations and then combine their reports at full committee meetings. This model of organisation was intended to ensure that people from all parts of the sub-County were consulted about the handover of Natukobenyo. The former chair of the CDDC emphasised the importance of incorporating perspectives from across the sub-County.

This issue of focusing on people close to the camps – we discussed this but feared it could produce a lot of animosity. If needs aren’t met, people will use force to meet them. When I was a county counsellor, there were a lot of tensions, because the host community wanted access to food aid and water. It was those living far from the camps who felt left out and took to violent methods to get their “services” from UNHCR. (Phone Interview, October 2020)

By incorporating the viewpoints of both proximal and distant people, the CDDC hoped to avoid resentment among those living far from the settlement. This also reflected the view that the land in Natukobenyo was communal and belonged to everyone. Similarly, official accounts from the TCG depict land in Turkana as a commons resource, in which ‘people are free to graze and settle in any area of their choice’ (TCG 2013: 19). On the one hand, this recognition of communal rights stands against privatisation and enclosure of pasturelands. It recognises that pastoralists require the flexibility to move their livestock throughout the territory according to the availability of seasonal vegetation. However, this particular conception of ‘communal land’ overlooks the complex social processes through which people negotiate access to different kinds of land use. Unlike conventional common property regimes, in which resources are governed by strict rules applied to clearly defined territories and groups, pastoralist land use is characterised by unbounded territories, contested group membership and negotiated access (Behnke 2018). Rather than a single coherent system, land can be governed through a ‘complex mosaic’ of rules, which are applied unevenly across scales and in regard to different resources (Robinson 2019).

From this perspective, then, Natukobenyo did not belong to any one group, but was a place where different groups had multiple, overlapping and at times contested claims. For those with cattle—who spend most of the year in the highlands of Oropoi, Nawounitos or even further west in Uganda—Natukobenyo was a source of wet season graze to which herders could return following the rainy season. In actuality, they have not returned to Natukobenyo in large numbers since 2007 because the area no longer supports the species of grass that cattle prefer. Nonetheless, herders in the FGD in Nawounitos emphasised their collective rights to places like Natukobenyo. In principle, this extends to all people of the *Ng’ilukumong* territorial section, who occupy a vaguely defined territory starting near Kakuma and extending westward to the border.

But residents of Natukobenyo emphasised a more exclusive claim to the area, referring to it as the place where they lived, tended goats and cultivated gardens along the seasonal streams. For this group, the handover of Natukobenyo was about more than lost pasture; it also meant that they had to leave their homes and abandon their gardens. Technically, they were permitted – and even encouraged – to continue cultivating in the area, a point that was made clear during a series of public outreach events convened by UNHCR and local chiefs. And indeed, some of the long-time Natukobenyo residents. But by July 2017, most people had chosen to move beyond the boundaries of the settlement, citing a loss of vegetation, increases in livestock theft, depletion of indigenous plant cover, and fear of insecurity at night. At the 2017 FGD that I conducted in Natukobenyo, some attendees pointed to a nearby stream where the remnants of thorn fences marked their old gardening sites.^1^ In their view, they should have had the loudest voice in the *barazas*—the meetings called by the CDDC—but the inclusion of everyone from the wider sub-County left them drowned out by the crowd.

Similarly, participants in the Lokwamor FGD explained that they used to graze their goats along *Ayanae Esikiriait*, *Kangura*, *Ayanae Elelea*, and *Ayanae Angidapala*, all streams in Natukobenyo.

Even after the refugees arrived in Natukobenyo, our animals still grazed inside the settlement. Until they became frightened by the iron-roofed houses. We had some people from this place [Lokwamor] who were living in Natukobenyo at that time, but they returned here after the refugees were settled in that land. (Turkana Woman, Lokwamor FGD, August 2017)

Because the TCG defined Natukobenyo as a commons resource, the community dialogues encompassed the entire population of Turkana West sub-County. While admirably inclusive, this approach overwrote differences in the impacts experienced by particular individuals and groups, who drew on different combinations of goods—grazelands, gardens, residences—and would be affected differently by the settlement.

This is not to suggest that merely narrowing the geographic band of recognised households would be an adequate alternative. The relevant unit of analysis is the extended family, within which resources are shared. Most families traverse large geographical distances. Women in the Natukobenyo FGD described how their gardens sustained not only their local households, but also their relatives who had migrated west with the cattle. At the FGD in Nawounitos, herders pointed out that they had left behind and occasionally sought support from extended family members who were living in Natukobenyo.

One word that encompasses this tension between the collective land claims of the wider population with the exclusive claims of particular people is *ere* (pl. *ng’ireria*). The word is shared by several closely related and even mutually intelligible languages in the region, and its meaning varies according to differences in socio-ecological context. Amongst Karamojong agro-pastoralists to the west in Uganda, *ere* refers to permanent settlements organised by agnatic descent, and it approximates a claim to private property. Young Karamojong herders migrate away from the *ere* with their animals during the dry season and then return at the start of the rains, when grass becomes available around the settlement. However, in much of Turkana, the more arid environment has made permanent settlements impractical. Therefore, an *ere* in Turkana may refer not to a village, but to the general area where a herder returns during the rains. This is a more tenuous attachment to place that is less easily reduced to private property rights. It refers to collective notions of territorial belonging. During the FGD in Nawounitos, one herder explained that Natukobenyo—along with Locileta, Lokwamor, Abaat and even Kakuma—are the *ng’ireria* of the entire Ng’ilukumong territorial section, where they can access wet season pastures during the long rains (*akiporo*).

In Turkana, exclusive claims to an *ere* are negotiable and may be contested. They are not inalienable like private property rights, but are rather contingent on the present residence of one’s relatives as well as the graves of ancestors. Pastoralists negotiate their access to wet season grazelands in the presence of both elders and administration chiefs, who attempt to ensure that the distribution of herds will not result in overgrazing. Some herders have priority to move to a particular area because they leave behind a segment of their household, including those who are too young or elderly to be helpful in the cattle camps, as well as some women who can cultivate sorghum and maize along the small seasonal rivers. The presence of their relatives in a place may be taken as evidence that it is their *ere.*

Because they are so contested and open to negotiation, claims to an *ere* do not provide a concrete identification of ownership and are often dismissed by formal authorities attempting to arbitrate land claims. The solution of the TCG was to avoid individual or household-based land claims to Natukobenyo and focus on broad communal possession of land for everyone in Turkana West. But this geographical approach to inclusion failed to account for differences in people’s relation to—and reliance upon—the Natukobenyo site and left some groups feeling disenfranchised by the process. An alternative would be to focus less on land claims as bundles of territorialised rights and instead address the particular ways that different groups use the various resources on that land.

#### Oppidan bias

For people in Turkana West, the dialogue process conducted by the CDDC was a means of advocating for greater ‘host community’ benefits from the UNHCR. Through the ToE, the CDDC successfully brought the concerns of a broad swathe of Turkana society—including urban entrepreneurs, urban working class and rural herders—to the table. However, support for pastoralists was gradually dwarfed by investments in the agricultural and retail sectors. This section considers how the structure and composition of the CDDC may have contributed to the marginalisation of pastoralists’ concerns.

The CDDC was formed through a participatory process in which two local leaders were nominated from each ward throughout Turkana West sub-County. The process for nominating representatives was broadly inclusive, but inevitably the individuals most suited for a committee role fit a particular profile: urban, formally educated professionals, many of whom had previously worked for the government or NGOs. This demographic is sometimes described as the ‘elite’ class in Turkana. Some non-literate individuals were included as representatives, but not among the core 7-member committee of the CDDC. Elites are well-positioned to function as an interface between members of the public and international organisations like UNHCR. But they are also less directly involved in the everyday activities of pastoralism, and their attention is often oriented to the infrastructural and entrepreneurial projects implemented by the major development organisations.

For this reason, the reliance on elite mediation produces an oppidan bias toward a vision for development that is urban and agricultural and focused on the cash economy. Even if the community consultation process was broadly inclusive, that content is ultimately communicated through the core membership of the CDDC, who are the only individuals with whom UNHCR directly engages. This bias is not lost on the wider population; as one woman living on the periphery of the Kalobeyei Settlement explained:

Those people in town (*ng’itunga lua arek*) are the ones who benefit. They get jobs with the big organisations, or working for the government, or starting businesses in the settlement. We *ng’iraiya* just watch as our livestock are finished by drought. We just survive on the charcoal and firewood that we sell to the refugees. But these people in town have opened their shops. (Turkana Woman, Natukobenyo, Interview, September 2018)

This statement was repeated along similar lines by many interviewees in Kalobeyei. The reference to *ng’iraiya* rather than ‘herders’ (*ng’ikeyokok*) points to a social distinction that is more complicated than pastoralists vs non-pastoralists, incorporating social differentiation based on various sources of cultural capital such as formal education, linguistic competency and familiarity with urban life (Rodgers 2020b). It suggests not only that pastoralists have been marginalised, but that urban elites have enjoyed much of the benefit of the settlement. Looking at spending by humanitarian agencies in Kalobeyei, this is undeniable. For each month of 2018, a total of about 500,000 USD was received as restricted cash transfers by refugees in the Kalobeyei Settlement. The restrictions meant that these transfers could only be spent at 45 shops contracted by World Food Programme (WFP) (Betts et al. 2019). This gave a small number of refugee and Kenyan entrepreneurs a large captive market. In mid-2019, WFP began the transition to unrestricted transfers, which makes this food retail market open to more businesses. Regardless, rural herders without entrepreneurial skills are unlikely to benefit from this as much as urban residents.

Turton (2003) describes ‘two principal characters’ who often play an important intermediatory role in pastoralists’ engagement with states: the Politician and the Priest. Whereas the Priest is a customary leader whose role is integrally grounded in the herding community—often a respected seer, diviner or prophet—the Politician is a formally educated individual somewhat removed from the day-to-day activities of pastoralism. Even if they are in an appointed rather than an elected position—and thus are not ‘politicians’ in the usual sense—their suitability for the role requires that they hold the respect and trust of the communities with whom they work, as well as the linguistic ability to communicate with them. Moreover, the Politician’s formal education and professional experiences endow them with the cultural capital required to engage effectively with state institutions as well as international NGOs.

This cultural capital, however, can also generate biases. Versed in the conventional narratives of development, urban elites are more likely to commit to a vision of progress based on economic growth, urbanisation and even agricultural development. This is not an example of ‘elite capture’, in which influential individuals divert and profit from international resources intended for the community. In many cases, the problem is that elite intermediaries earnestly—and often with good intentions—buy into the vision of development presented by international institutions and their funders.

While the problem of elite bias is shared by most representative democracies, it is especially stark in places like Turkana, where the cultural capital of formal education and professional experience creates such extreme differences in interests, preferences and epistemes between an elite minority and a pastoralist majority. And if pastoralist leaders—as embodied in the trope of the Priest—do not see urban elites as legitimate representatives, they may attempt to circumvent their authority. This is illustrated in the case of the *agata* event described above, which preceded UNHCR’s withdrawal from Lokwamor and Locileta. During my interviews, former members of the CDDC recalled that the *agata* ceremony was done behind their back.

Moreover, the problem of oppidan bias does not fall solely on the shoulders of elite representatives such as the members of the CDDC. As noted above, the CDDC did recognise pastoralists’ concerns when drafting the ToE. However, the ToE was a non-binding document akin to a memorandum of understanding. It provided principles and priorities that could inform—but not determine—the course of the intervention in Kalobeyei. The ToE ultimately fed into the composition of KISEDP, a policy document specifying not principles but concrete projects and objectives. There is some support to pastoralism under Component 6 of KISEDP, which is titled ‘Agriculture, Livestock and Natural Resource Management’. It includes a short list of strategies to ‘increase livestock production and productivity’, such as establishing water points along migration corridors, reseeding of pasturelands and seasonal restocking of herds. However, to date, there has been little actual investment in these strategies. Moreover, some of the listed strategies, such as digitising the livestock branding system and ‘modernising’ slaughter systems, are low on the list of priorities of herders. The key indicators for Component 6 of KISEDP all pertain to agriculture, with no indicators by which to assess support for pastoralists.

The problem, in this case, was not only that urban professionals led the community engagement process, but that the actual engagement with the public was reduced to an initial phase preceding the ToE. As the ToE was operationalised in the form of KISEDP, UNHCR and the TCG took the lead, with some input by the CDDC. But there was almost no involvement by the wider Turkana population as these plans evolved, and so pastoralist priorities gave way to the oppidan model of development. This is perhaps best captured by a short section in the introduction of the KISEDP document, which begins by noting that pastoralism is practised by approximately 65% of the county population, but then depicts trends of change and transition:

Poorer households are either ‘dropping out’ of pastoralism or choosing alternative livelihood options, relying more heavily on food sources such as food aid, payments in kind, crops and wild foods, and to rely on (*sic*) safety nets, crop sales, self-employment and casual employment as income sources. In particular, many Turkana young men and women no longer only want to become pastoralists and they often seek to combine a nomadic lifestyle with an education and/or employment opportunity. (UNHCR 2018: 10)

The suggestion is that pastoralism is a way of life that is no longer viable and is now on the wane. This corresponds to familiar narratives of sedentarisation and transition that have influenced development in pastoralist areas for decades (Krätli et al. 2015). An alternative framing that emphasises the continued significance of pastoralism *despite* increasing social stratification, urbanisation and livelihood diversification would be equally consistent with the cited data.

## Conclusion

Compared to the allocation of land for the Kakuma camp in the early 1990s, the UNHCR’s efforts at community engagement over the Kalobeyei Settlement are commendable and show an increased recognition of Turkana people’s rights. The efforts of the CDDC brought the concerns of various people from across the socio-economic spectrum to the attention of the UNHCR and TCG, who have responded with a plan that orients humanitarian funding to both refugee protection and local development. However, despite efforts to foster an inclusive process, several problems and misunderstandings ultimately resulted in the marginalisation of pastoralist voices.

Reflecting on the community engagement process for the Kalobeyei Settlement highlights several lessons for UNHCR’s engagement efforts in Kakuma, as well as other organisations implementing large-scale projects in pastoralist areas.

The first lesson pertains to land. Discussions over changes in land use should attend to the diverse impacts on different groups. Inclusivity entails that all relevant stakeholders are given a chance to express their views and that particular groups are not disenfranchised during the process. But inclusivity does not necessarily require that all participation is equal; in some cases, the voices of certain stakeholders should be given more weight, based on such factors as the specific form of land use, level of vulnerability or the degree to which they are affected by the proposed intervention. Despite the communal nature of grazing rights in Turkana, people living close to a site are likely to be affected in particular ways by an intervention. This does not mean that a smaller geographic radius will suffice for community engagement, which should also recognise communal grazing rights for anyone in the local territorial sections. The approach adopted by Tullow Oil, which is to undertake ‘systematic engagement with various levels of stake-holders’, would better account for the multiplicity of ways that different groups rely on land (Mkutu et al. 2019: 242).

Pastoralist land tenure may not adhere to a clear-cut system so much as ad hoc  negotiation processes. This seem incompatible with the more juridical approaches to property ownership upon which national legal systems rely. However, customary institutions can adjust to new circumstances, if community leaders are involved and given ‘a sense of ownership in the institution-building process’ (Haller et al. 2016: 412). In the short term, an approach to community dialogue that distinguishes between differences in land use and concomitant differences in the potential impact of disruptive interventions might yield more nuanced and just forms of compensation and benefit-sharing. As suggested by the contested notion of belonging embodied in the term *ere*, the customary mechanisms used among pastoralists to govern access to territories and resources are not perfectly compatible with statutory land regulation.

It would also be useful for future community engagement to distinguish negotiations about compensation—which often pit local stakeholders with competing claims against one another—from development dialogues aiming at broader public goods. As Herrera et al. note, ‘the success of participation often relies on… the acceptance of a common arena and objectives that could benefit the entire community’ (2014:4). Rather than promising development-as-compensation, which seems to fuel unrealistic expectations for ‘host benefits’, local people should have been directly compensated for lost access to land and resources *before* discussions about development plans began.

The second lesson pertains to the risks of relying too heavily on ‘elite’ (i.e. urban professional) gatekeepers. It is now widely recognised that ostensibly participatory procedures can veil processes of elite-driven privatisation, a risk that has been identified in communal land reform more generally (Gargule and Lengoiboni 2020: 342). But even where elite intermediaries act in good faith, they may introduce an ‘oppidan bias’ into development policy. While such gatekeepers provide a convenient interface between external development actors and local populations, their perspectives are shaped by the values and interests of the urban, educated demographic to which they belong. The lifestyles of most urban representatives separate them from the daily realities experienced by pastoralists (Songok et al. 2011). Community concerns that resonate with their own economic positions—access to employment, contracts and business opportunities—are more likely to be amplified, while the concerns of herders are attenuated.

One solution is to incorporate a more diverse range of stakeholders into dialogue committees. Aside from authority figures—who are usually men—committees should also ensure inclusion of female, youth and pastoralist representatives. While this may be contested by men accustomed to patriarchal rules for public dialogue, more inclusive participatory processes can be supported with trained facilitation, sustained commitment from institutional stakeholders, and sufficient funding (Herrera et al. 2014: 4). Language barriers can be overcome with real-time translation assistance, allowing a broader array of people to engage directly in dialogue.

The final lesson learned from the Kalobeyei experience is that UNHCR – or any agency implementing a large-scale dryland intervention – must have a clear strategy for community engagement. Looking ahead, improving processes for community dialogue will be crucial to achieving local legitimacy for future development interventions and extractive operations in Turkana, as well as meeting the legal requirement for public participation. While I do not recommend that UNHCR adopts Tullow’s particular approach to host community engagement, a more explicit policy on direct community engagement would be useful. In the recently published *Operational Guidance on Accountability to Affected Populations*, ‘affected populations’ are defined as ‘people of concern’ under UNHCR’s mandate, i.e. ‘asylum seekers, returnees, refugees, stateless, and internally displaced persons’ (2020: 5). ‘Host communities’ are only briefly mentioned at the end of the policy: ‘Inclusion of the host community is critical for effective protection, assistance and solutions programming, including to avoid tensions and competition for resources’ (UNHCR 2020b: 17). But there is no consideration of land issues, resource governance or development-related policies. This lack of attention to host communities seems out of touch with UNHCR’s commitment to an area-based approach or its recognition of the burdens facing people in refugee-hosting areas.

In developing a host community engagement strategy, UNHCR may draw upon some of the established procedures of the World Bank, with whom they have partnered in the development of KISEDP. For example, the World Bank policy note on community engagement recommends that implementing agencies should drive their own community engagement process: ‘although government agencies and civil society can provide an entry point into communities, their own interests and limited capacity can sometimes compromise the process’ (World Bank 2018: 1). While UNHCR does occasionally convene townhall meetings and outreach events with members of the Turkana host population, this is largely on an *ad hoc* basis. Usually, host community engagement is outsourced to the TCG, while UNHCR coordinates communication with refugee communities.

While elected government officials do possess the formal legitimacy of the democratic model, authority in Turkana is recognised amongst a more diverse array of administrative and customary leaders. Formal and customary structures are intertwined, but they are not completely consolidated into a unified system. The contested nature of authority is a long-standing feature of governance structures where communal land and resources are involved (Behnke 2018). Further research that attends to the nuances of negotiated authority processes and hybrid governance structures would better inform the design of inclusive community dialogue processes. This is important because if the dialogue leading to the handover of Natukobenyo has left some individuals feeling left out—especially influential community leaders—then the legitimacy of activities undertaken by the UNHCR may be challenged in the future.

## Data Availability

Not applicable

## Abbreviations

UNHCR: United Nations High Commissioner for Refugees
EPRDF: Ethiopian People’s Revolutionary Democratic Front
FGD: Focus group discussion
LWF: Lutheran World Federation
NRC: Norwegian Refugee Council
CDDC: Community Dialogue & Development Committee
TCG: Turkana County Government
ToE: Terms of Engagement
KISEDP: Kalobeyei Integrated Socio-Economic Development Plan
WFP: World Food Programme

## Footnotes

1 More recently, the Food and Agriculture Organisation and the World Food Programme have partnered to develop irrigated farming projects to be shared by refugees and hosts. These initiatives have yielded modest harvests. But some locals continue to lament that clearance of local vegetation has left less browse for goats and camels.
